# Differential Gene Expression and DNA Methylation in the Risk of Depression in LOAD Patients

**DOI:** 10.3390/biom12111679

**Published:** 2022-11-12

**Authors:** Suraj Upadhya, Daniel Gingerich, Michael William Lutz, Ornit Chiba-Falek

**Affiliations:** Division of Translational Brain Sciences, Department of Neurology, Duke University Medical Center, Durham, NC 27710, USA

**Keywords:** differential DNA methylation, differential gene expression, depression, late-onset Alzheimer’s disease

## Abstract

Depression is common among late-onset Alzheimer’s Disease (LOAD) patients. Only a few studies investigated the genetic variability underlying the comorbidity of depression in LOAD. Moreover, the epigenetic and transcriptomic factors that may contribute to comorbid depression in LOAD have yet to be studied. Using transcriptomic and DNA-methylomic datasets from the ROSMAP cohorts, we investigated differential gene expression and DNA-methylation in LOAD patients with and without comorbid depression. Differential expression analysis did not reveal significant association between differences in gene expression and the risk of depression in LOAD. Upon sex-stratification, we identified 25 differential expressed genes (DEG) in males, of which *CHI3L2* showed the strongest upregulation, and only 3 DEGs in females. Additionally, testing differences in DNA-methylation found significant hypomethylation of CpG (cg20442550) on chromosome 17 (log_2_FC = −0.500, *p* = 0.004). Sex-stratified differential DNA-methylation analysis did not identify any significant CpG probes. Integrating the transcriptomic and DNA-methylomic datasets did not discover relationships underlying the comorbidity of depression and LOAD. Overall, our study is the first multi-omics genome-wide exploration of the role of gene expression and epigenome alterations in the risk of comorbid depression in LOAD patients. Furthermore, we discovered sex-specific differences in gene expression underlying the risk of depression symptoms in LOAD.

## 1. Introduction

Late-onset Alzheimer’s Disease (LOAD) is a heterogeneous disease with comorbid clinical symptoms. Most pronounced are the neuropsychiatric symptoms (NPS) including depression that show high prevalence among LOAD patients [1,2,3,4,5,6,7]. In addition, it was demonstrated that LOAD biomarkers, such as cerebrospinal fluid (CSF) Aβ_42_ and CSF total and phosphorylated tau, are associated with depression trajectories in LOAD [8]. However, the genetic architecture underpinning the onset and heterogeneity of NPS in LOAD has been understudied, and there are only a few publications, including our previous work [9,10]. In prior work, we elucidated the shared genetic architecture between major depressive disorder (MDD) and LOAD. We reported common genetic pathways, such as immune and inflammatory pathways, between MDD and LOAD [9]. Additionally, we constructed a polygenic risk score (PRS) that was shown to predict depression onset within LOAD patients, resulting in moderate predictability [10]. Collectively, these studies indicated that genetic factors contribute to comorbid depression in LOAD.

Alteration of gene expression and mechanisms dysregulating gene expression were suggested to play a prominent role in the genetics underlying LOAD pathogenesis. Differential gene expression has been widely reported in LOAD [11,12,13], with studies uncovering differentially expressed genes (DEG) in bulk brain tissues [12] and within different brain cell types [11] cross-sectionally and throughout disease progression. Moreover, DNA-methylation, an epigenetic mechanism of regulation of gene expression, has also been studied in the context of LOAD. For example, De Jager et al. found 71 significant CpGs, while a recent epigenome-wide meta-analysis by Zhang et al. identified 3751 CpGs associated with LOAD [14,15]. These differential methylated regions have implicated several genes previously linked to LOAD. Similarly, the role of differential gene expression and DNA-methylation was also studied in depression. Several studies showed transcriptomic changes in MDD, implicating glutaminergic, dopaminergic, immune system, and inflammatory pathways [16,17,18,19]. However, Cole et al. found no significant differential gene expression when looking at peripheral blood mononuclear cells (PBMC) samples [20]. With regard to DNA-methylation in depression, a meta-analysis using 11,256 subjects found three CpG sites significantly differentially methylated among people with depression compared to those without [21]. However, these studies looked at differences between patients and healthy controls and did not provide insights into the molecular characterization of LOAD comorbid with depression. Characterization of the transcriptomic and epigenetic landscapes in LOAD patients with and without comorbid depression will improve the genetic and mechanistic understanding of depression heterogeneity in LOAD.

This work applied differential gene expression and DNA-methylation analyses to study the endophenotype of depression in LOAD using a subset of the Religious Orders Study and Rush Memory and Aging Project (ROSMAP) dataset. This study extends prior work for the prediction of individuals with LOAD who are at greater risk of developing depression [9,10]. Collectively these studies aim to characterize the heterogeneity of depression in LOAD and further expand the field of heterogenous symptoms in LOAD by providing a guideline to study other endophenotypes of LOAD.

## 2. Materials and Methods

### 2.1. Study Cohort

The sample was taken from a subset of the Religious Orders Study and Rush Memory and Aging Project (ROSMAP) dataset [14,22,23,24]. ROS has enlisted nuns and brothers since 1994. MAP has recruited individuals from the northern Illinois region since 1997. Both studies were run by the same investigators using similar data collection techniques. Thus, the results from both were comparable. A LOAD case was ascertained based on the final consensus cognitive diagnosis variable (cogdx = 4 or 5). The LOAD diagnosis was made by a consensus of neurologists and neuropsychologists reviewing post-mortem clinical data. A depression case was determined using the clinical diagnosis variable (r_depres) [25]. The value from this variable was derived from criteria from the Diagnostic and Statistical Manual of Mental Disorders, 3rd Edition, Revised [26], the patient’s responses to questions from the Diagnostic Interview Schedule, and a clinical interview of the patient. The variable had four endpoints: highly probably, probable, possible, and not possible depression. If the person had any of the first three options at any time in the study duration, the individual was deemed a depression case.

The total sample with DNA-methylation data contained 603 subjects, with 252 LOAD cases with depression data. Of the 252 LOAD cases, there were 79 depression cases and 173 controls. Of the total sample, there were 145 depression cases and 458 controls. A sub-sample of this data was also needed, as all subjects did not have both DNA-methylation and RNA-seq data. Thus, only those with both datasets were included in the RNA-seq analysis. For the LOAD only RNA-seq sample, there was a total of 166 subjects, with 50 and 116 depression cases and controls, respectively. In the full RNA-seq sample, of the total of 424 subjects, 99 and 325 were cases and controls, respectively. Table 1 displays the demographics of the samples used in the analysis.

### 2.2. Differential Expression

RNA-seq data from bulk cell dorsolateral prefrontal cortex tissue (Broadman area 46) was retrieved from Synapse (Synapse ID: syn8691134). Raw counts were taken of 61,775 transcript IDs. Transcripts with at least 2 counts in 10% of depression cases within both cohorts, the LOAD sample and the full sample, were kept. This filtering resulted in 38,053 and 38,028 transcripts used for differential expression analysis for the LOAD and the full sample cohorts, respectively. The R package DESeq2 was used to perform the normalization and differential expression analysis [28]. Briefly, DESeq2 develops a generalized linear model (GLM) for each gene and then employs Wald tests to evaluate the differential expression. The statistical models included age at death (age_death), sex (msex), Braak stage (braaksc), and post-mortem interval (PMI), study number (ROS or MAP), RNA integrity number (RIN), number of ribosomal bases, number of aligned reads, batch, and three genotyping principal components (PC) as covariates calculated by Mostafavi et al. [29]. The *p* values are corrected for multiple comparison testing using the Benjamini and Hochberg method [30]. Differential expression analysis was done on the two cohorts, LOAD samples only and the full sample. To assess the effects of covariates on gene expression, multiple regressions of PC1 with all covariates were done, with logistic regressions done with categorical variables and linear regressions done with numeric variables. To analyze sex-specific effects, the LOAD and full cohorts were stratified by sex and the analyses were repeated.

### 2.3. Differential DNA-Methylation

The DNA-methylation data of the sample was generated previously from dorsolateral prefrontal cortex (Broadman area 46, same region as the RNA-seq data) using the Illumina HumanMethylation450 Beadchip Array (450K) [14] and retrieved from Synapse (Synapse ID: syn3157275). The 450k array profiled a total number of 420,132 CpGs sites. The data was reported as beta-values, where this value is calculated as the ratio of the methylated CpGs intensity over the total sum of both unmethylated and methylated intensities. The values fall between 0 and 1, which indicates no or complete methylation, respectively [31]. Beta-values provide an intuitive framework for biological interpretation, with 0–100% methylation values. M-values, the log transform of beta values, were found to be more effective for differential analysis [31]. However, we used beta values for subsequent analyses to remain consistent with the original report [14].

The DNA-methylation data had been normalized and batch corrected, as detailed in [14]. The differential DNA-methylation analysis was performed using GLM. Two models were made. The control model was made of only the covariates: sex, age at death, PMI, and Braak stage. The test model was composed of the covariates and each CpGs site. Each respective test model, one for each CpG, was compared to the control mode using a likelihood ratio test. This test was done to assess the impact of the CpGs site on the depression diagnosis. Multiple comparison testing was done for the differential methylation analyses, with the adjusted *p*-value calculated using the Benjamini and Hochberg method [30]. Both differential DNA-methylation analyses were done on two cohorts: (1) only LOAD patients and (2) the full sample. In addition, we stratified the cohorts by sex and repeated the analyses separately in the males only and females only groups.

## 3. Results

### 3.1. Differential Expressed Genes Are Associated with Depression Symptoms in LOAD Patients in a Sex-Specific Manner

**Figure 1:**
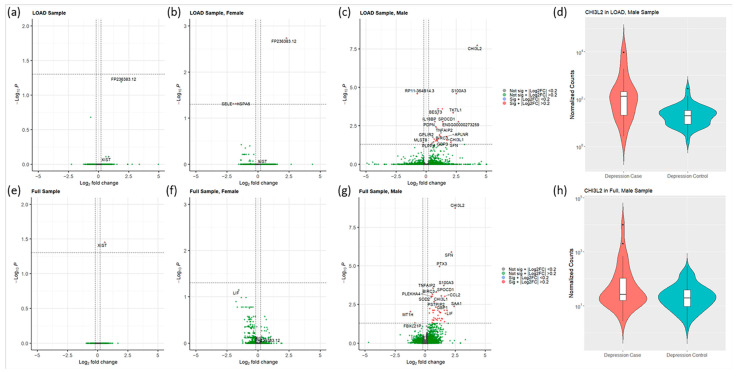
Differential gene expression analysis identified sex-specific expression in depression. Volcano plots of the (**a**) LOAD sample, (**b**) female subset of LOAD sample, and (**c**) male subset of LOAD sample. There were no DEGs in the LOAD sample, while the sex-stratified analysis detected 3 and 25 DEGs within the female and male samples, respectively. (**d**) *CHI3L2* was significantly upregulated within the male, LOAD sample illustrated by the violin plot (log_2_FC = 4.248, *p* = 1.84 × 10^−8^). Volcano plots of the (**e**) full sample, (**f**) female subset of full sample, and (**g**) male subset of full sample. *XIST* was significantly upregulated in the full sample (log_2_FC = 0.594, *p* = 0.035), while the sex-stratified analysis demonstrated no DEGs in the female sample while the male sample had 44 DEGs. (**h**) *CHI32* was upregulated in the full, male sample demonstrated by the violin plot (log_2_FC = 2.481, *p* = 1.74 × 10^−9^). Adjusted *p*-value cutoff of 0.05 and Log_2_FC cutoff of 0.2 was used for the volcano plots.

Differential gene expression analysis using the LOAD sample did not identify any significant differential expressed genes (DEGs) associated with depression symptoms. However, *FP236383.12*, encoding a non-coding RNA, showed a suggestive trend of increased expression (log_2_FC = 1.863, log_2_FCSE = 0.389 *p* = 0.064; Figure 1a and Table 2). We then tested associations with confounding factors that might affect mRNA levels. We found that PC1 is significantly associated with sex as a biological covariate (*p* = 0.025; Figure S1A). Thus, we stratified the LOAD sample by sex and repeated the analyses in the male and female groups separately to identify sex-specific differential gene expression associations with depression symptoms in LOAD. The analysis of the male group identified 25 DEGs with *CHI3L2,* encoding a protein involved in carbohydrate binding, showing the strongest upregulation effect (log_2_FC = 4.248, log_2_FCSE = 0.588, *p* = 1.84 × 10^−8^; Figure 1c and Table 2). In addition, *CHI3L1,* another member of the carbohydrate binding family, showed significantly increased expression in male LOAD cases with depression vs. LOAD only (log_2_FC = 1.736, log_2_FCSE = 0.401, *p* = 0.030; Table 2). In the female LOAD sample, 3 DEGs were found to be significantly associated with depression in LOAD (Figure 1b and Table 2). While *FP236383.12* demonstrated a robust effect in females (log_2_FC = 2.289, log_2_FCSE = 0.419, *p* = 0.002), it has been retired in the most recent genome assembly; thus, we did not further explore the association with this gene.

In the full sample, *XIST* was the only identified DEG (log_2_FC = 0.594, log_2_FCSE = 0.121, *p* = 0.035; Figure 1e and Table S1). Noteworthy, *XIST* gene was also upregulated in the LOAD sample but did not reach statistical significance (log_2_FC = 0.841, log_2_FCSE = 0.212, *p* = 0.777). Sex-stratified analysis of the full sample resulted in 44 DEGs in the male group and none in the female group (Figure 1f,g and Table S1). We would like to note that in the male group of the full sample we also identified significant upregulation of *CHI3L2* (log_2_FC = 2.481, log_2_FCSE = 0.329, *p* = 1.74 × 10^−9^; Figure 1h) and *CHI3L1* (log_2_FC = 1.342, log_2_FCSE = 0.257, *p* = 0.001) similarly to the LOAD cohort.

### 3.2. Differential DNA-Methylation Sites Are Associated with Depression Symptoms in LOAD Patients

**Figure 2:**
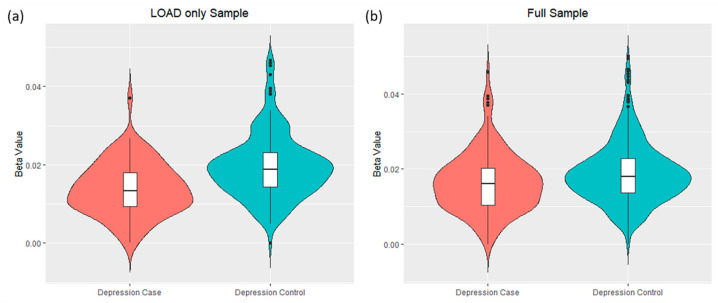
Differential DNA-methylation identified lower methylation levels at one CpG site within CpG Island positioned at Chr17:73,511,016-73,513,176 (measured by probe cg20442550) in LOAD with depression compared to LOAD only. Beta-values represent methylation on a 0–1 scale, with 1 indicating 100% methylation. Beta-values for this CpG site were plotted against depression case–controls in the (**a**) LOAD only and (**b**) full sample, i.e., LOAD and normal control. This CpG site showed significantly lower levels of DNA-methylation only with the LOAD sample ((**a**) beta = −116.6, log_2_FC = −0.500, *p* = 0.004).

**Figure 3:**
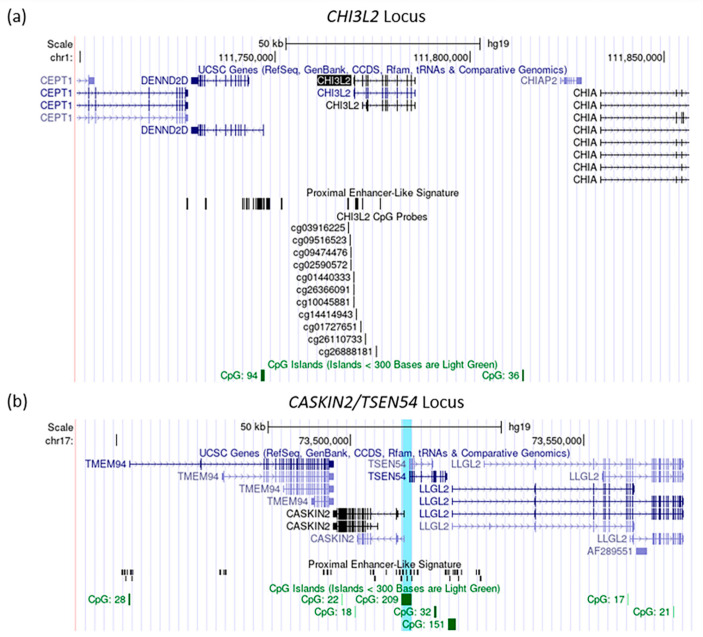
Genomic views of (**a**) CpG island sites across the *CHI3L2* locus and flanking genomic regions (**b**) The vacancy surrounding CpG Island located at Chr17:73,511,016-73,513,176 (contains cg20442550 in Illumina 450K array). Highlighted in light blue the is location of the CpG island with 209 CG dinucleotide counts that had one probe differentially methylated between LOAD patients with vs. without depression. The CpG island is located between *CASKIN2* and *TSEN54.* Images were made using the UCSC genome browser hg19/GRCh37 [32], and included the following tracks: Base position, UCSC Genes, CpG Islands, and ENCODE cCRE (Liftover was used to convert between CRCh38 and GRCh37).

Differential DNA-methylation analysis identified significant hypomethylation associated with depression in LOAD of one probe that covers a CpG island located on Chr17:73,511,016-73,513,176 (cg20442550, beta = −116.6, log_2_FC = −0.500, *p* = 0.004, Figure 2 and Table S2). This probe is positioned in the intergenic region between CASKIN2 and TSEN54 (Figure 3b). The other 17 probes covering this CpG island did not show significant changes in DNA-methylation levels (Table S3). Another probe in the CpG island located on Chr19:41,035,100-41,035,440, near SPTBN4, showed a borderline statistically significant association with depression (cg02795700, beta = −22.7, log_2_FC = −0.616, *p* = 0.053). When differential DNA-methylation analysis was repeated in the full sample, no significant CpG sites were found (Table S4). Next, we performed DNA-methylation analysis stratified by sex and did not identify any significant CpG sites in either the male or female groups.

### 3.3. Integration Analysis of the Gene Expression and the DNA-Methylation Associations with Depression Symptoms in LOAD

Integration of the transcriptomic and DNA-methylomic association (results outlined in Section 3.1 and Section 3.2) did not identify overlap in the identified genomic regions (Figure 3). There were no significant probes in the regions of any of the 28 DEGs from the sex-stratified LOAD analysis. For example, the eleven tested CpGs sites across the CHI3L2 genomic locus showed no significant differential DNA-methylation in either the LOAD, male subsample or the full, male sample (Tables S5 and S6, respectively). Additionally, the probes within the CpG island 23 kb upstream of CHI3L2 also did not show significant differential DNA-methylation (Figure 3a, Tables S7 and S8). Since the DNA-methylation analysis includes only probes covering autosomal chromosomes, we could not perform the integration analysis for XIST, which is located on chromosome X.

The CpG site measured by probe cg20442550 is 1500bp upstream of the transcription start site of CASKIN2 and 200 bp from the transcriptional start site of TSEN54 (Figure 3b). Neither CASKIN2 (log_2_FC = −0.016, *p* = 0.999), a protein-binding protein, nor TSEN54 (log_2_FC = −0.024, *p* = 0.999), a subunit of a tRNA endonuclease complex [33,34] were differentially expressed in association with depression symptoms in the LOAD sample or the sex-stratified analysis.

## 4. Discussion

LOAD is characterized by multiple clinical symptoms [35,36,37,38], with NPS, including depression, showing high prevalence [1,2,3,4,5,6,7]. Prior work has explored the common genetics underlying MDD and LOAD, using genetics to predict those at greater risk of developing depression [9,10]. However, candidate genes and regulatory mechanisms underlying comorbid depression in LOAD have been understudied, and the transcriptomics and DNA-methylomics characteristics of depression heterogeneity in LOAD remain largely unknown. Here, for the first time, we performed a comprehensive investigation to explore differences in both gene expression and DNA-methylation within LOAD patients with compared to without depression. We further studied sex-specific molecular differences of comorbid depression in LOAD.

Differences in prevalence between men and women have been reported in multiple neurological diseases. For example, autism spectrum disorders and early onset schizophrenia are more prevalent among men, while females are affected by anxiety disorders and multiple sclerosis to a greater degree [39]. Most relevant to this study, it has been suggested that depression has a higher prevalence in females [40], while men may have more severe forms of reported depression [41]. Similarly, LOAD prevalence is also affected by sex, with a greater risk for females [42,43]. Furthermore, a meta-analysis reported a higher risk for comorbid NPS, including depression, in female LOAD patients, while male LOAD patients had more severe apathy [44]. Our results shed new light on differential gene expression associated with sex-dependent depression risk in LOAD.

Our differential gene expression analysis found sex-specific differential expression in depression comorbid with LOAD, with males having a greater number of DEGs than females. *CHI3L2*, a carbohydrate-binding protein [45], was identified as the top significantly upregulated DEG in the male LOAD group. *CHI3L2* has been linked with cognitive impairment in AD, with its inflammatory roles cited [46]. Inflammatory pathways have been implicated in many neurological disorders, including neurodegeneration and NPS [47,48,49]. Of those pathways, *CHI3L2* was found to be associated with IBA1-mediated microglial activation and PECAM1-mediated blood–brain barrier (BBB) changes, inflammatory processes partly contributing to neurodegeneration [50]. With regard to alterations of the BBB, Feng et al. reviewed evidence demonstrating BBB changes in depression and schizophrenia, highlighting a potential role of the inflammatory pathway and *CHI3L2* in the pathology of both LOAD and MDD [48]. Moreover, a study testing antidepressant treatment found that individuals who responded to treatment saw a significant decrease in *CHI3L2* expression, which was found in a male sample and replicated in a clinical-like sample [51]. The authors suggested *CHI3L2* and other implicated genes as biomarkers for antidepressant treatment [51]. Our work may further explain this finding and provide evidence to support *CHI3L2* as a marker for depression in LOAD. *CHI3L2* was also significantly upregulated in the full, male sample, although with a smaller fold change. This further suggests *CHI3L2* involvement in depression in general.

In the overall full sample, we found *XIST* to be significantly upregulated, with a suggestive trend in the LOAD sample. *XIST* codes for long non-coding RNA *Xist*, X inactive specific transcript, which is involved in the inactivation of one X chromosome in females [52]. *XIST* has been implicated in neuropsychiatric disorders [53] and LOAD [54,55,56], to which our results provide additional support.

Integrative analysis did not detect any statistically significant correlations between DNA-methylation and gene expression. Of note, the DNA-methylation coverage did not include sex chromosomes. Therefore, we could not test for a correlation between DNA-methylation and gene expression for the XIST gene. In addition to DNA-methylation, other mechanisms participate in gene regulation, for example, histone modifications [57,58], alteration in transcription factor binding sites [59,60], miRNAs [57,61], and alternative splicing [62,63]. In this regard, these regulatory mechanisms have previously been found to be associated with MDD and LOAD. For example, histone deacetylases (HDAC) have been associated with MDD and LOAD. *Hdac5* expression was lowered in the nucleus accumbens in an MDD mouse model [57], while HDAC2 and HDAC6 levels were elevated in LOAD [58]. HDAC2 reduces the acetylation of genes tied to learning and memory, and HDAC6 plays a role in increasing phosphorylated tau [58]. Another transcription regulator is the alteration of transcription factor binding sites, which have been found to be altered by SNPs found in both MDD and LOAD GWAS [59,60]. In terms of post-transcription regulation, many miRNAs have been linked to pathways of both diseases, with miR-132 lowering BDNF expression in MDD [57] and tied to both tau and amyloid pathology in LOAD [61]. Additionally, alternate splicing, which leads to different forms of mRNA, was noted in the serotonin-1a receptor in depression, where a miRNA target site is spliced out. This form was reduced in MDD [62]. In LOAD, many notable genes, *APOE4*, *PSEN1*, *PSEN2,* and *APP*, experience alternate splicing [63]. Regulatory mechanisms may also be sex-dependent, as was found with chromatin accessibility in LOAD [64].

DNA-methylation analysis identified a differentially methylated CpG on chromosome 17 within the LOAD only sample. Nonetheless, we did not detect differential expression of the nearest genes, *CASKIN2* and *TSEN54*. It is important to note that this CpG site is hypomethylated in controls with 1.95% DNA-methylation levels (LOAD with no depression). Thus, we interpret that the further decrease in DNA-methylation levels observed in LOAD with depression cases may not have a functional impact and therefore has no detectable effect on the expression of the nearby genes. Furthermore, other probes colocalized within the same CpG island as the significant probe had no significant differential methylation.

Another study explored differential DNA-methylation in MDD using a similar subset of ROSMAP [65]. They found 7 differentially methylated CpG sites, with 4 clustering within the same CpG island in the YOD1 gene locus. Our results did not replicate their findings. One possible explanation is the differences in the design of the case–control groups. While the cases group in our study was defined as individuals with depression diagnosis at any time in the study (N = 145), the case–control design in Hüls et al. defined cases as subjects with an MDD diagnosis at baseline. This resulted in fewer cases (N = 30) than our case sample [65]. Moreover, the tested questions were different. Hüls et al. aimed to find differential DNA-methylation in MDD that are not confounded by Alzheimer’s [65], while here we studied LOAD comorbid with depression.

Our study has several limitations. First, we used data generated by the Illumina 450k DNA methylation array, which has several limitations. Many CpGs are non-variable within a specific tissue, which leads to the inclusion of many non-variable CpGs when making comparisons in a single tissue. As the methylation sample was taken from a specific tissue, dorsolateral prefrontal cortex, the non-variable CpGs may contribute to the more stringent multiple comparison testing [66]. Thus, more CpGs may be highlighted by addressing this concern. Similarly, with many CpGs clumped together, a potential multiple collinearity problem arises [67]. As SNPs are clumped due to linkage disequilibrium in PRS calculation, a similar method could be implemented to address multiple collinearities of the methylation CpGs. Additionally, the small sample size and homogeneity of the sample may limit our ability to discover DEGs and differentially methylated CpGs within the two analyses. A larger sample size will provide increased power to detect smaller effect sizes for differentially methylated CpGs.

Our study provides insights into the pan and sex-specific epigenetic and transcriptomic factors underlying depression in LOAD. To the best of our knowledge, this is the first and most comprehensive multi-omics and sex-specific interrogation of the comorbidity of depression with LOAD. Thus, our findings expand the field by advancing our current genomic understanding of comorbid depression in LOAD in general and in a sex-specific manner. Our pipeline could be implemented to study the risk and heterogeneity of NPS phenotypes and other comorbid conditions in LOAD.

## 5. Conclusions

We presented a new pipeline to study differential omics of endophenotypes of LOAD, applying it to comorbid depression in LOAD. Our approach identified differential expression of *CHI3L2* among depression cases that was stronger within the LOAD only cohort. Interestingly, *CHI3L* has been previously associated with LOAD [46,50]. Additional omics data and experimental approaches will be warranted to determine the regulatory mechanisms underlying *CHI3L2*’s differential expression, sex-specificity, and mechanistic role in the context of depression and LOAD.

## Figures and Tables

**Table 1:** Sample Demographics.

	RNA-Seq Sample LOAD Only	RNA-Seq Sample All	Methylation Sample LOAD Only	Methylation Sample All
**Subjects**	166	424	252	603
**Depression Cases/Controls**	50/116	99/325	79/173	145/458
**Mean age at death (SD)**	91.0 (6.1)	88.5 (6.6)	873.80 (3.66.5)	86.583.0 (4.56.5)
**Percent Female**	66%	63%	66%	63%
**Mean Years of Education (SD)**	16.6 (3.4)	16.5 (3.5)	16.3 (3.5)	16.4 (3.5)
**APOEe4 Count:**				
**0**	102	309	160	435
**1**	6	111	86	159
**2**	4	4	6	9
**Mean PMI ^1^ in hours (SD)**	6.8 (4.1)	7.0 (4.9)	6.8 (4.7)	7.5 (6.0)
**Percent Braak Stage ≥ 4**	67%	52%	69%	52%
**Mean MMSE ^2^ at Last Visit (SD)**	13.6 (8.7)	21.6 (8.9)	12.8 (8.6)	20.9 (9.3)

^1^ PMI—Post-mortem Interval. ^2^ MMSE—Mini-Mental State Examination [27].

**Table 2:** Sex-Stratified LOAD Differential Expression Analysis Results.

	Male		Female		LOAD	
Gene	Log_2_FC	*p*	Log_2_FC	*p*	Log_2_FC	*p*
APLNR	1.652	**0.018**	−0.333	0.999	0.084	0.999
BEST3	0.997	**0.0003**	−0.093	0.999	0.213	0.999
BIRC3	0.995	**0.025**	−0.193	0.999	0.173	0.999
CHI3L1	1.736	**0.03**	−0.447	0.999	0.286	0.999
CHI3L2	4.246	**1.84 × 10^−8^**	−0.715	0.999	1.191	0.999
ENSG00000232306.1	−8.116	**0.005**	NA	NA	−0.441	0.999
ENSG00000273259.2	2.669	**0.002**	−1.424	0.999	−0.175	0.999
FP236383.12	−0.556	0.999	2.289	**0.002**	1.863	0.064
GBP3	0.922	**0.034**	−0.06	0.999	0.26	0.999
GBPI	1.343	**0.0003**	−0.522	0.999	0.09	0.999
GPLIR2	0.673	**0.026**	−0.04	0.999	0.116	0.999
HSPA6	0.868	0.999	−1.79	**0.049**	−0.617	0.999
IL18BP	0.727	**0.002**	−0.188	0.999	0.072	0.999
LRRC55	0.78	**0.034**	−0.087	0.999	0.129	0.999
MLST8	−0.243	**0.043**	0.06	0.999	−0.001	0.999
PDPN	0.829	**0.005**	−0.225	0.999	0.045	0.999
PLEKHA4	0.946	**0.0007**	−0.241	0.999	0.082	0.999
PLPP4	0.619	**0.038**	−0.111	0.999	0.095	0.999
RP11-364B14.3	−0.745	**2.67 × 10^−5^**	0.096	0.999	−0.084	0.999
S100A3	2.496	**2.67 × 10^−5^**	−0.306	0.999	0.678	0.999
SELE	1.935	0.688	−1.988	**0.049**	−0.794	0.999
SFN	2.049	**0.041**	−0.508	0.999	0.39	0.999
SLAMF8	1.155	**0.017**	−0.826	0.999	0.117	0.999
SPOCD1	1.368	**0.002**	−0.3	0.999	0.095	0.999
TIMP1	0.87	**0.03**	−0.456	0.999	−0.037	0.999
TKTL1	2.234	**0.0006**	−0.583	0.999	0.362	0.999
TNFAIP2	1.098	**0.014**	−0.185	0.999	0.235	0.999
VAMP5	0.452	**0.048**	−0.119	0.999	0.041	0.999

## Data Availability

ROSMAP resources and data can be requested at: https://www.radc.rush.edu, accessed on 18 January 2022. ROSMAP datasets (clinical, transcriptomic, and methylomic) were downloaded on 18 January 2022. The R script used for the differential DNA-methylation analysis can be found at: https://github.com/suraj-upad/differential-dna-methylation, accessed on 18 January 2022.

## Supplementary Materials

The following supporting information can be downloaded at: https://www.mdpi.com/article/10.3390/biom12111679/s1, Figure S1: Covariate associations with genotyping principal component; Table S1: Sex-stratified analysis results of the full sample; Table S2: Top CpG probes from LOAD only sample; Table S3: All CpG probes within CpG Island located at Chr17:73,511,016-73,513,176 from LOAD only sample; Table S4: Top CpG probes from the full Sample; Table S5: CpG probes within *CHI3L2* locus from LOAD, male sample; Table S6: CpG probes within *CHI3L2* locus from the full, male sample; Table S7: Probes within CpG island upstream of *CHI3L2* locus from LOAD, male only sample; Table S8: Probes within CpG island upstream of *CHI3L2* locus from full, male only sample.
