# Supplementary material for: Differential Gene Expression and DNA Methylation in the Risk of Depression in LOAD Patients
Source: Biomolecules. 2022 Nov 12;12(11):1679. doi: 10.3390/biom12111679 (PMC9687527; doi:10.3390/biom12111679)
Supplement: Supplementary file 1 [file biomolecules-12-01679-s001.zip › biomolecules-1975631-supplementary.pdf]

Article

Differential Gene Expression and DNA Methylation in the Risk of Depression in LOAD Patients

Suraj Upadhya, Daniel Gingerich, Michael William Lutz and Ornit Chiba-Falek*

Division of Translational Brain Sciences, Department of Neurology, Duke University Medical Center, Durham, NC 27710, USA;
* Correspondence: Ornit Chiba-Falek; E-mail: o.chibafalek@duke.edu; Tel.: +919-681-8001

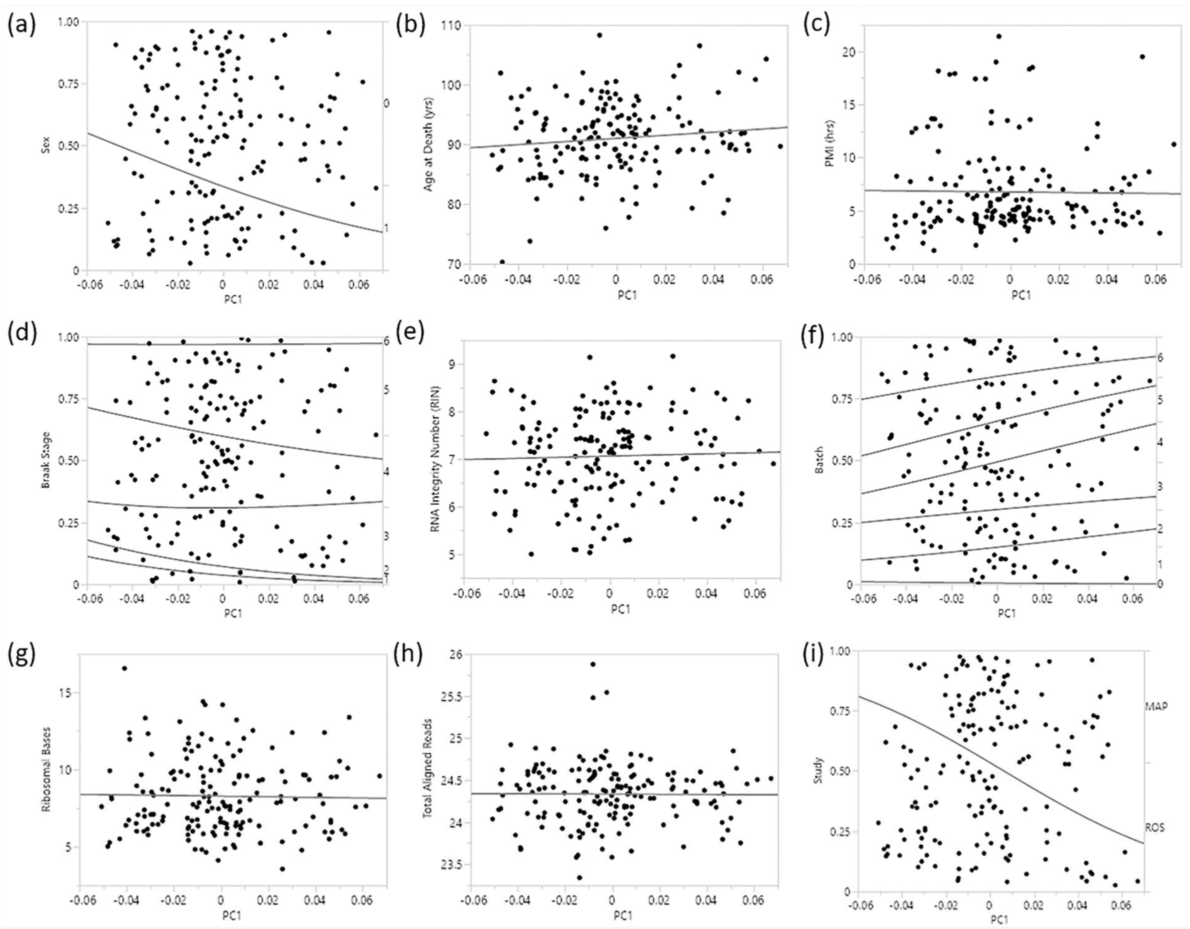

Figure S1: Covariate associations with genotyping principal component. Logistic or linear regressions were done for each covariate and the first principal component (PC1). Covariates included in the model were (a) sex, (b) age at death, (c) post-mortem interval (PMI), (d) Braak stage, (e) RNA integrity number, (f) batch, (g) number of ribosomal bases, (h) total aligned reads, and (i) study, either ROS or MAP. Only sex (p=0.025) and study (p=0.007) had significant

associations with PC1. As sex was the only biological covariate with a significant association with genotyping PCs, the sample was stratified by sex for further analysis.

Table S1: Sex-stratified analysis results of the full sample

	Male		Female		Full	
	Log2FC	P[1]	Log2FC	P[1]	Log2FC	P[1]
ADGRE1	1.678	**0.012**	-0.764	0.959	0.18	0.99995
BCL2A1	1.285	**0.035**	-0.757	0.747	0.087	0.99995
BIRC3	0.616	**0.0007**	-0.153	0.999	0.163	0.99995
CCL2	1.632	**0.0009**	-0.656	0.999	0.213	0.99995
CCL4	1.611	**0.04**	-0.633	0.999	0.548	0.99995
CD44	1.27	**0.011**	-0.479	0.999	0.275	0.99995
CHI3L1	1.342	**0.0009**	-0.663	0.759	0.138	0.99995
CHI3L2	2.481	**1.74E-09**	-1.059	0.449	0.604	0.99995
CXCL3	0.661	**0.011**	-0.391	0.759	0.03	0.99995
CXCL8	1.384	**0.025**	-0.197	0.999	0.438	0.99995
EBI3	0.767	**0.008**	-0.272	0.999	0.172	0.99995
EPHA1	0.353	**0.011**	0.005	0.999	0.112	0.99995
FBXL21P	-0.849	**0.049**	-0.206	0.999	-0.412	0.99995
FSTL3	0.378	**0.006**	-0.12	0.999	0.068	0.99995
GBP1	0.887	**0.005**	-0.71	0.282	0.01	0.99995
GPR84	0.929	**0.029**	-0.717	0.168	-0.057	0.99995
ICAM1	1.043	**0.008**	-0.337	0.932	0.15	0.99995
IFITM3	0.588	**0.046**	-0.254	0.999	0.066	0.99995
IL1B	1.415	**0.027**	-0.906	0.439	0.072	0.99995
IL1RN	1.589	**0.007**	-0.614	0.999	0.533	0.99995
IL4I1	0.71	**0.032**	-0.334	0.999	0.083	0.99995
LIF	1.805	**0.008**	-1.544	0.071	-0.092	0.99995
LINC01686	0.704	**0.032**	-0.354	0.999	0.098	0.99995
MK280592	1.178	**0.025**	-0.03	0.999	0.435	0.99995
MT1H	-1.222	**0.009**	0.486	0.999	0.108	0.99995
NFKB2	0.582	**0.031**	-0.239	0.999	0.094	0.99995
PLEKHA4	0.508	**0.0009**	-0.307	0.959	0.027	0.99995
PRKAR1B-AS1	0.651	**0.008**	-0.244	0.999	0.08	0.99995
PSTPIP2	0.747	**0.002**	-0.167	0.999	0.191	0.99995
PTX3	1.181	**1.10E-05**	-0.692	0.31	0.147	0.99995
RGS16	1.01	**0.032**	-0.439	0.999	0.151	0.99995
S100A3	1.545	**0.0002**	-0.434	0.999	0.494	0.99995
SAA1	2.403	**0.004**	-1.669	0.168	0.761	0.99995
SECTM1	1.064	**0.016**	-0.968	0.104	-0.021	0.99995
SERPINE1	1.052	**0.025**	-0.407	0.999	0.173	0.99995
SFN	2.193	**1.24E-06**	-1.05	0.439	0.391	0.99995
SLAMF8	0.638	**0.008**	-0.559	0.747	0.057	0.99995

SOD2	0.582	**0.0009**	-0.217	0.999	0.072	0.99995
SPIB	1.176	**0.009**	-0.646	0.999	0.276	0.99995
SPOCD1	0.905	**0.0005**	-0.69	0.598	-0.008	0.99995
TIMP1	1.248	**0.011**	-0.774	0.31	-0.01	0.99995
TNFAIP2	0.662	**0.0003**	-0.364	0.759	0.093	0.99995
TNFAIP3	0.721	**0.023**	-0.277	0.999	0.158	0.99995
TNFAIP6	0.841	**0.025**	-0.302	0.999	0.192	0.99995
XIST	1.226	0.058	0.009	0.999	0.594	**0.039**

[1] Adjusted p-value using the Benjamini-Hochberg method

Table S2: Top CpG probes from LOAD only sample

CpG	Chromosome	Nearby Genes	Estimate	P	Adjusted P	Log2FC
**cg20442550**	17	CASKIN2; TSEN54	-111.314	5.16E-08	0.022	-0.500
**cg02795700**	19	SPTBN4; SPTBN4	-22.732	2.51E-07	0.053	-0.616

Table S3: All CpG probes within CpG Island located at Chr17:73,511,016-73,513,176 from LOAD only sample

CpG	Estimate	P	Adjusted P	Log2FC
**cg01803810**	-12.622	0.108	0.999994	-0.077
**cg02077420**	9.028	0.573	0.999994	0.028
**cg04121983**	6.273	0.037	0.999994	0.119
**cg05606039**	-8.945	0.324	0.999994	-0.025
**cg07724304**	5.262	0.239	0.999994	0.014
**cg07897831**	-32.216	0.073	0.999994	-0.286
**cg09173924**	-0.990	0.910	0.999994	-0.013
**cg14349867**	-81.257	0.006	0.999994	-0.589
**cg14955495**	0.365	0.990	0.999994	-0.046
**cg15862680**	14.706	0.529	0.999994	0.081
**cg17901382**	3.110	0.412	0.999994	0.004
**cg20442550***	-116.586	1.03E-08	0.004	-0.500
**cg21038819**	1.456	0.761	0.999994	1.23E-05
**cg23932769**	-5.824	0.694	0.999994	-0.044
**cg24220383**	4.061	0.767	0.999994	0.016
**cg25506900**	-8.991	0.491	0.999994	-0.013
**cg25654301**	1.443	0.933	0.999994	0.016
**cg27037648**	8.803	0.024	0.999994	0.093

*Significant CpG probe

Table S4: Top CpG probes from the full Sample

CpG	Chromosome	Nearby Genes	Estimate	P	Adjusted P	Log2FC
**cg13389382**	9	C9orf167	9.974	4.88E-07	0.205	0.049

Table S5: CpG probes within *CHI3L2* locus from LOAD, male sample

CpG	Estimate	P	Adjusted P	Log2FC
**cg26110733**	-11.128	0.485	0.927	-0.003
**cg26366091**	0.958	0.871	0.987	0.007
**cg26888181**	2.128	0.913	0.992	-0.0003
**cg01440333**	-3.574	0.719	0.968	-0.003
**cg01727651**	-25.876	0.012	0.682	-0.027
**cg02590572**	-13.450	0.463	0.922	-0.003
**cg03916225**	-7.029	0.181	0.822	-0.042
**cg09474476**	-1.429	0.519	0.934	-0.020
**cg09516523**	-7.046	0.599	0.949	-0.006
**cg10045881**	-6.727	0.385	0.902	-0.004
**cg14414943**	-7.097	0.515	0.933	-0.005

Table S6: CpG probes within *CHI3L2* locus from the full, male sample

CpG	Estimate	P	Adjusted P	Log2FC
**cg26110733**	0.164	0.986	0.9998	-0.0001
**cg26366091**	-3.637	0.336	0.976	-0.014
**cg26888181**	-0.974	0.928	0.9998	-0.0007
**cg01440333**	-0.315	0.961	0.9998	-0.0004
**cg01727651**	-17.912	0.004	0.733	-0.019
**cg02590572**	1.986	0.847	0.9997	0.001
**cg03916225**	-1.553	0.615	0.995	-0.014
**cg09474476**	-0.017	0.991	0.9998	-0.005
**cg09516523**	-8.132	0.363	0.978	-0.005
**cg10045881**	-1.653	0.741	0.999	-0.002
**cg14414943**	-4.680	0.521	0.991	-0.004

Table S7: Probes within CpG island upstream of *CHI3L2* locus from LOAD, male only sample

CpG	Estimate	P	Adjusted P	Log2FC
**cg00619207**	-5.129	0.472	0.923	-0.008
**cg06125456**	8.819	0.091	0.767	0.035
**cg07318155**	-46.191	0.014	0.691	-0.105
**cg08663890**	-4.626	0.554	0.941	-0.009
**cg08864105**	0.540	0.914	0.992	0.005
**cg09826895**	-2.901	0.759	0.973	-0.003
**cg11071762**	-14.842	0.630	0.955	-0.039
**cg13547268**	-71.794	0.035	0.714	-0.188
**cg14519917**	-0.800	0.911	0.992	-0.0005
**cg16354651**	-31.767	0.184	0.824	-0.129
**cg19268695**	-6.398	0.366	0.896	-0.011
**cg19269039**	-7.541	0.206	0.834	-0.020
**cg20317872**	-5.739	0.339	0.888	-0.016
**cg20970369**	-6.054	0.290	0.870	-0.018
**cg21220708**	0.147	0.983	0.998	0.032
**cg22609277**	-18.269	0.032	0.710	-0.200
**cg23631538**	-6.703	0.310	0.878	-0.018
**cg24641737**	-3.489	0.560	0.942	-0.012
**cg25722212**	-6.174	0.505	0.931	-0.019
**cg26115312**	7.127	0.346	0.890	0.170
**cg26518580**	-9.125	0.055	0.737	-0.065

Table S8: Probes within CpG island upstream of *CHI3L2* locus from full, male only sample

CpG	Estimate	P	Adjusted P	Log2FC
**cg00619207**	2.266	0.619	0.995	0.005
**cg06125456**	5.187	0.108	0.891	0.019
**cg07318155**	-6.129	0.611	0.995	-0.016
**cg08663890**	-6.952	0.194	0.940	-0.012
**cg08864105**	1.965	0.558	0.993	0.008
**cg09826895**	1.454	0.795	0.9996	0.0005
**cg11071762**	-2.106	0.918	0.9997	-0.005
**cg13547268**	-18.524	0.371	0.979	-0.059
**cg14519917**	2.306	0.617	0.995	0.008
**cg16354651**	-12.177	0.412	0.983	-0.077
**cg19268695**	-1.046	0.815	0.9997	-0.002
**cg19269039**	-1.698	0.679	0.998	-0.005
**cg20317872**	0.785	0.830	0.9997	0.002
**cg20970369**	0.406	0.915	0.9997	0.002
**cg21220708**	-0.777	0.868	0.9997	-0.024
**cg22609277**	-2.081	0.696	0.998	-0.029
**cg23631538**	1.610	0.678	0.998	0.009

**cg24641737**	0.157	0.969	0.9998	-0.001
**cg25722212**	1.265	0.833	0.9997	0.005
**cg26115312**	3.008	0.507	0.989	0.068
**cg26518580**	-6.225	0.071	0.853	-0.026
